# General health literacy, COVID-19-related health literacy, and protective behaviors: evidence from a population-based study in Japan

**DOI:** 10.3389/fpubh.2023.1208815

**Published:** 2023-09-18

**Authors:** Keiko Murakami, Shinichi Kuriyama, Hideki Hashimoto

**Affiliations:** ^1^Tohoku Medical Megabank Organization, Tohoku University, Sendai, Japan; ^2^Graduate School of Medicine, Tohoku University, Sendai, Japan; ^3^Department of Health and Social Behavior, School of Public Health, The University of Tokyo, Tokyo, Japan; ^4^Department of Disaster Public Health, International Research Institute of Disaster Science, Tohoku University, Sendai, Japan

**Keywords:** COVID-19, health literacy, healthy lifestyle behaviors, Japan, protective behaviors

## Abstract

**Introduction:**

Health literacy (HL) can be regarded as a key element of non-pharmaceutical interventions used in emergency responses. The present study aimed to determine the associations of combinations of general HL and COVID-19-related HL with COVID-19 protective behaviors and healthy lifestyle behaviors.

**Methods:**

A questionnaire survey was conducted from December 2020 to January 2021 among residents in Japanese metropolitan areas. Valid responses were received from 1,443 residents. The levels of HL were categorized into four groups: low level in both HLs (reference), high level in general HL only, high level in COVID-19-related HL only, and high level in both HLs. The total scores of eight COVID-19 protective behaviors were dichotomized into low and high adherence. Healthy lifestyle behaviors included healthy and balanced diet, adequate sleep, and regular exercise. Poisson regression analyses were conducted to examine the associations between the HL groups and high adherence to COVID-19 protective behaviors.

**Results:**

High level in COVID-19-related HL only was associated with high adherence to COVID-19 protective behaviors [prevalence ratio (PR), 1.25; 95% confidence interval (CI), 1.09–1.45], while high level in general HL only was associated with healthy and balanced diet (PR, 1.49; 95% CI, 1.04–2.13), adequate sleep (PR, 1.46; 95% CI, 1.02–2.10), and regular exercise (PR, 2.00; 95% CI, 1.29–3.13). High level in both HLs showed the highest prevalence of high adherence to COVID-19 protective behaviors and healthy lifestyle behaviors.

**Conclusion:**

These findings indicate that COVID-19-related HL and general HL can both be considered to enhance protective behaviors.

## Introduction

Since the beginning of the coronavirus disease 2019 (COVID-19) pandemic, governments have implemented both pharmaceutical and non-pharmaceutical measures to curb the spread of the virus (1). While efforts to develop pharmaceutical interventions for COVID-19 are under way, non-pharmaceutical interventions still play an important role in the control of the pandemic (2–4). Therefore, promoting motivation and adherence to non-pharmaceutical preventive behavioral recommendations presents an important public health challenge. In addition, healthy lifestyle behaviors play an important role in maintaining physical and mental health during the COVID-19 pandemic, as well as during ordinary times (5, 6).

Health literacy (HL) can be regarded as a key element of non-pharmaceutical interventions used in emergency responses (7, 8). HL has been defined as people’s knowledge, motivation, and competence to access, understand, appraise, and apply health information to make judgements and decisions in everyday life concerning healthcare, disease prevention, and health promotion to maintain or improve their quality of life (9). Therefore, HL is critical for informed decision-making and empowers people and communities (10). There is accumulating evidence that people with higher levels of HL are more likely to adhere to COVID-19 protective behaviors (11–20). However, these studies focused mainly on general HL. This type of HL may be inadequate for assessing responses to the COVID-19 pandemic because HL is context-dependent and therefore requires different forms of cognitive processing (21). It is possible that people with generally acceptable HL skills may still face HL challenges in certain contexts. To the best of our knowledge, no studies have examined the associations between combinations of general HL and COVID-19-related HL and COVID-19 protective behaviors. Furthermore, it remains unclear whether HL is differently associated with COVID-19 protective behaviors and healthy lifestyle behaviors.

Considering the above circumstances, we aimed to determine the associations of combinations of general HL and COVID-19-related HL with COVID-19 protective behaviors and healthy lifestyle behaviors in Japan. Unlike other countries, Japan has relied heavily on voluntary behavioral changes and cooperation, because the public health measures implemented by the Japanese government to mitigate COVID-19 do not have penalties for non-adherence. Therefore, we hypothesized that HL is a key determinant of adherence to COVID-19 protective behaviors.

## Materials and methods

### Study population

Data were obtained from the Japanese Study of Stratification, Health, Income, and Neighborhood (J-SHINE), which has been described elsewhere (22). The first-wave survey was conducted in four municipalities in and around the greater Tokyo metropolitan area between October 2010 and February 2011. Among 13,920 residents aged 25–50 years who were probabilistically selected from the residential registry in each of these four municipalities, survey staff members were able to contact 8,408 residents and received valid responses from 4,317 residents. The survey participants were re-contacted in 2012 and 2017 (the second and third waves) (23). The fourth-wave survey was conducted between December 2020 and January 2021 (24). Among 1,638 residents who responded, 97.0% responded online and 3.0% responded offline. We analyzed 1,443 participants with no missing values for any variables used in the analyses. The Research Ethics Committee of The University of Tokyo, Graduate School of Medicine approved the survey procedure of the J-SHINE (Approval No. 3073). The J-SHINE Data Management Committee approved the secondary use of the data, with personally identifiable information deleted to ensure confidentiality.

### General HL and COVID-19-related HL

General HL was measured using the Communicative and Critical Health Literacy (CCHL) scale for the first-wave questionnaire (25). This scale was developed and validated in Japan to assess communicative and critical HL (26). Communicative HL refers to the capacity to collect necessary and appropriate information to support one’s actions and communicate the information to others, while critical HL refers to one’s capacity to critically evaluate the quality of available information and select appropriate information for use in decision-making (26). Participants were asked whether they could do the following: collect health-related information from various sources; extract the information they want; understand and communicate the obtained information; consider the credibility of the information; and make decisions based on the information, in the context of health issues. Each item was rated on a five-point Likert scale, ranging from 1 (strongly disagree) to 5 (strongly agree). The scores of these five responses were summed and divided by the number of items to determine a total score (theoretical range: 1–5), with higher scores indicating greater HL (25). The Cronbach’s alpha value of the scale was 0.83, and this was not improved by the deletion of any specific item.

COVID-19-related HL was measured based on the questionnaire in the “Survey tool and guidance” edited by the World Health Organization Regional Office for Europe included in the fourth-wave survey (27). Participants were asked how easy or difficult they found it to: find the information they need related to COVID-19; understand information about what to do if they think they have COVID-19; judge if the information about COVID-19 in the media is reliable; understand restrictions and recommendations of authorities regarding COVID-19; follow the recommendations on how to protect themselves from COVID-19; understand recommendations about when to stay at home from work/school, and when not to; and understand recommendations about when to engage in social activities, and when not to. Each item was rated on a five-point Likert scale, ranging from 1 (very difficult) to 5 (very easy). The scores of these seven responses were summed and divided by the number of items to determine a total score (theoretical range: 1–5), with higher scores indicating greater HL. The Cronbach’s alpha value of the scale was 0.85, and this was not improved by the deletion of any specific item. According to the process-knowledge model of HL (21), HL can be synergistically acquired through processing capacity and knowledge. Furthermore, knowledge can offset limitations in processing capacity. We can argue that the general HL measure we relied on (CCHL) measures the perceived capacity for processing health information rather than knowledge. In contrast, the context-specific HL measure we adopted (COVID-19-related HL) measures domain-specific knowledge and the capacity to process the optimal responses for controlling COVID-19 infection. Adopting this theory frame, we could then hypothesize that high scores in COVID-19-related HL (specific knowledge-based HL) would result in a marked likelihood of adoption of COVID-19 protective behavior, regardless of the level of general HL (processing capacity-based HL). In contrast, high general HL is likely to be associated with non-specific healthy lifestyle behaviors.

HL was categorized into four groups, after dichotomizing each HL by the median score (25): low level in both HLs (low general HL and low COVID-19-related HL; reference), high level in general HL only (high general HL and low COVID-19-related HL), high level in COVID-19-related HL only (low general HL and high COVID-19-related HL), and high level in both HLs (high general HL and high COVID-19-related HL).

### COVID-19 protective behaviors

Participants were asked whether they had performed the COVID-19 protective behaviors during the past 1 month, based on the protective measures against COVID-19 recommended by the World Health Organization: wearing a mask outside their home; staying at home as much as possible; avoiding gatherings; avoiding the use of public transportation; regularly and thoroughly cleaning their hands with an alcohol-based hand rub or washing them with soap and water; covering their mouth and nose with their bent elbow or tissue when they cough or sneeze; disinfecting surfaces frequently, especially those that are regularly touched; and maintaining at least a 2 m distance between themselves and others when outdoors. Each item was rated on a five-point Likert scale as never (1), almost never (2), sometimes (3), almost always (4), or always (5). The scores of these eight responses were summed and divided by the number of items to determine a total score (theoretical range: 1–5), with higher scores indicating adherence to more protective behaviors. The Cronbach’s alpha value of the scale was 0.77, and this was not improved by the deletion of any specific item. High adherence to total protective behaviors was defined as a total score greater than the median score of 4.

### Healthy lifestyle behaviors

Three types of health-related behaviors were measured: healthy and balanced diet, adequate sleep, and regular exercise (5). Each item was rated on a five-point Likert scale as never, almost never, sometimes, almost always, or always. Responses to each item were dichotomized as low adherence (never, almost never, sometimes, almost always) and high adherence (always) to healthy lifestyle behaviors.

### Covariates

As covariates, we chose age, sex, education level (high school or lower, college, university or higher), annual household income (≤4.99, 5.00–9.99, ≥10.00 million Japanese yen, unknown/refusal), work status (working, not working), self-rated health, and psychological distress. Self-rated health was measured using the question “How would you describe your health during the past 1 month?” Responses were dichotomized as good health (excellent, very good, good) and poor health (fair, poor) for the purposes of analysis. Psychological distress was measured using the K6 scale, which comprises six items assessing depressive moods and anxiety during the past 1 month on a five-point Likert scale ranging from 0 (none of the time) to 4 (all the time) (28, 29). A cut-off score of 5 was used to identify psychological distress (29, 30).

### Statistical analysis

The characteristics of the participants were compared between the HL groups using the chi-square test. Poisson regression analyses with robust variance estimators were conducted to examine the associations between the HL groups and high adherence to COVID-19 protective behaviors. Prevalence ratios (PRs) and 95% confidence intervals (CIs) were calculated after adjustment for covariates. Low level in both HLs was set as the reference category. Similar analyses were conducted for healthy lifestyle behaviors.

All analyses were conducted with Stata 16.0 (StataCorp LP, College Station, TX, United States). For all analyses, a two-tailed *p-*value <0.05 was considered statistically significant.

## Results

Table 1 shows the characteristics of the participants. The prevalences of the HL groups were 35.8% for low level in both HLs, 15.1% for high level in general HL only, 26.1% for high level in COVID-19-related HL only, and 23.0% for high level in both HLs. Participants with high level in both HLs were more likely to be men, be highly educated, and have higher income, and less likely to have psychological distress.

**Table 1:** Characteristics of participants: the Japanese Study of Stratification, Health, Income, and Neighborhood (J-SHINE).

	General HL and COVID-19-related HL								*p*-value^a^
	Low level in both HL (*n* = 517)		High level in general HL only (*n* = 217)		High level in COVID-19-related HL only (*n* = 377)		High level in both HL (*n* = 332)		
Age, *n* (%)									0.043
35–44 years	170	(32.9)	62	(28.6)	134	(35.5)	86	(25.9)	
45–54 years	225	(43.5)	97	(44.7)	173	(45.9)	159	(47.9)	
55–61 years	122	(23.6)	58	(26.7)	70	(18.6)	87	(26.2)	
Women, *n* (%)	305	(59.0)	124	(57.1)	201	(53.3)	163	(49.1)	0.032
Educational attainment, *n* (%)									0.007
High school or lower	114	(22.1)	45	(20.7)	67	(17.8)	49	(14.8)	
College	191	(36.9)	70	(32.3)	136	(36.1)	101	(30.4)	
University or higher	212	(41.0)	102	(47.0)	174	(46.1)	182	(54.8)	
Annual household income, *n* (%)									<0.001
≤4.99 million Japanese yen	179	(34.6)	67	(30.9)	124	(32.9)	71	(21.4)	
5.00–9.99 million Japanese yen	184	(35.6)	78	(35.9)	156	(41.4)	134	(40.4)	
≥10.00 million Japanese yen	79	(15.3)	43	(19.8)	62	(16.4)	99	(29.8)	
Unknown/refusal	75	(14.5)	29	(13.4)	35	(9.3)	28	(8.4)	
Working, *n* (%)	451	(87.2)	196	(90.3)	335	(88.9)	296	(89.2)	0.63
Poor self-rated health, *n* (%)	51	(9.9)	23	(10.6)	39	(10.3)	30	(9.0)	0.92
Psychological distress, *n* (%)	266	(51.5)	97	(44.7)	140	(37.1)	120	(36.1)	<0.001

HL, health literacy.

a Obtained using the chi-square test.

Table 2 presents the associations between combinations of general HL and COVID-19-related HL and high adherence to COVID-19 protective behaviors. High level in COVID-19-related HL only, but not high level in general HL only, was associated with high adherence to COVID-19 protective behaviors; the multivariate-adjusted PRs were 1.25 (95% CI, 1.09–1.45) and 1.11 (95% CI, 0.93–1.34), respectively. High level in both HLs was associated with high adherence to COVID-19 protective behaviors; the multivariate-adjusted PR was 1.29 (95% CI, 1.11–1.50). Female sex, low income, and non-working status were associated with high adherence to COVID-19 protective behaviors, while age, education, self-rated health, and psychological distress were not associated with adherence to COVID-19 protective behaviors.

**Table 2:** Associations between combinations of general HL and COVID-19-related HL and high adherence to COVID-19 protective behaviors.

	High adherence /participants	(%)	Crude		Multivariate-adjusted^a^	
			PR (95% CI)		PR (95% CI)	
Total	646/1443	(44.8)				
*General HL and COVID-19-related HL*						
Low level in both HLs	207/517	(40.0)	1.00		1.00	
High level in general HL only	94/217	(43.3)	1.08	(0.90–1.30)	1.11	(0.93–1.34)
High level in COVID-19-related HL only	186/377	(49.3)	1.23	(1.06–1.43)	1.25	(1.09–1.45)
High level in both HLs	159/332	(47.9)	1.20	(1.03–1.40)	1.29	(1.11–1.50)
*Age*						
35–44 years	210/452	(46.5)	1.00		1.00	
45–54 years	298/654	(45.6)	0.98	(0.86–1.12)	0.99	(0.87–1.12)
55–61 years	138/337	(41.0)	0.88	(0.75–1.04)	0.90	(0.77–1.06)
*Sex*						
Men	220/650	(33.9)	1.00		1.00	
Women	426/793	(53.7)	1.59	(1.40–1.80)	1.50	(1.31–1.71)
*Educational attainment*						
High school or lower	119/275	(43.3)	1.00		1.00	
College	264/498	(53.0)	1.23	(1.05–1.44)	1.15	(0.98–1.34)
University or higher	263/670	(39.3)	0.91	(0.77–1.07)	0.96	(0.81–1.13)
*Annual household income*						
≤4.99 million Japanese yen	209/441	(47.4)	1.00		1.00	
5.00–9.99 million Japanese yen	252/552	(45.7)	0.96	(0.84–1.10)	0.99	(0.87–1.13)
≥10.00 million Japanese yen	106/283	(37.5)	0.79	(0.66–0.95)	0.80	(0.67–0.96)
Unknown/refusal	79/167	(47.3)	1.00	(0.83–1.20)	0.95	(0.79–1.14)
*Work status*						
Working	550/1278	(43.0)	1.00		1.00	
Not working	96/165	(58.2)	1.35	(1.17–1.56)	1.18	(1.02–1.36)
*Self-rated health*						
Good	578/1300	(44.5)	1.00		1.00	
Poor	68/143	(47.6)	1.07	(0.89–1.28)	1.06	(0.89–1.27)
*Psychological distress*						
No	372/820	(45.4)	1.00		1.00	
Yes	274/623	(44.0)	0.97	(0.86–1.09)	0.97	(0.86–1.10)

HL, health literacy; PR, prevalence ratio; 95% CI, 95% confidence interval.

a Adjusted for all other variables in the table.

Table 3 presents the associations between combinations of general HL and COVID-19-related HL and healthy lifestyle behaviors. High level in general HL only, but not high level in COVID-19-related HL only, was associated with healthy and balanced diet; the multivariate-adjusted PRs were 1.49 (95% CI, 1.04–2.13) and 1.33 (95% CI, 0.97–1.81), respectively. High level in both HLs was associated with healthy and balanced diet; the multivariate-adjusted PR was 2.09 (95% CI, 1.57–2.79). High level in general HL only, but not high level in COVID-19-related HL only, was associated with adequate sleep; the multivariate-adjusted PRs were 1.46 (95% CI, 1.02–2.10) and 1.29 (95% CI, 0.94–1.76), respectively. High level in both HLs was associated with adequate sleep; the multivariate-adjusted PR was 1.83 (95% CI, 1.36–2.47). High level in general HL only, high level in COVID-19-related HL only, and high level in both HLs were associated with regular exercise; the multivariate-adjusted PRs were 2.00 (95% CI, 1.29–3.13), 1.57 (95% CI, 1.04–2.37), and 1.82 (95% CI, 1.21–2.75), respectively. Psychological distress was associated with low adherence to all three healthy lifestyle behaviors.

**Table 3:** Associations between the combination of general HL and COVID-19-related HL and healthy lifestyle behaviors.

	Healthy and balanced diet			Adequate sleep			Regular exercise		
	%	PR (95% CI)		%	PR (95% CI)		%	PR (95% CI)	
Total	18.3			17.1			11.5		
*General HL and COVID-19-related HL*									
Low level in both HLs	12.6	1.00		12.4	1.00		7.2	1.00	
High level in general HL only	18.9	1.49	(1.04–2.13)	18.0	1.46	(1.02–2.10)	14.8	2.00	(1.29–3.13)
High level in COVID-19-related HL only	17.5	1.33	(0.97–1.81)	17.2	1.29	(0.94–1.76)	12.5	1.57	(1.04–2.37)
High level in both HLs	27.7	2.09	(1.57–2.79)	23.5	1.83	(1.36–2.47)	15.1	1.82	(1.21–2.75)
*Age*									
35–44 years	17.3	1.00		18.6			11.7		
45–54 years	18.7	1.07	(0.83–1.39)	17.4	0.94	(0.73–1.22)	11.3	0.90	(0.64–1.27)
55–61 years	19.0	1.08	(0.80–1.46)	14.2	0.76	(0.55–1.05)	11.6	0.90	(0.61–1.33)
*Sex*									
Men	15.9	1.00		14.6			11.9		
Women	20.3	1.30	(1.02–1.65)	19.0	1.31	(1.02–1.68)	11.2	0.89	(0.66–1.21)
*Educational attainment*									
High school or lower	12.4	1.00		12.4			8.0		
College	21.1	1.58	(1.11–2.26)	19.1	1.43	(1.00–2.05)	12.9	1.57	(0.99–2.51)
University or higher	18.7	1.42	(1.00–2.03)	17.5	1.33	(0.93–1.90)	11.9	1.29	(0.82–2.04)
*Annual household income*									
≤4.99 million Japanese yen	19.1			19.1			10.4		
5.00–9.99 million Japanese yen	17.6	0.86	(0.66–1.11)	16.1	0.78	(0.60–1.02)	11.2	0.96	(0.67–1.38)
≥10.00 million Japanese yen	22.3	0.97	(0.72–1.30)	19.1	0.84	(0.62–1.16)	15.9	1.32	(0.89–1.97)
Unknown/refusal	12.0	0.61	(0.39–0.97)	11.4	0.61	(0.39–0.97)	7.8	0.74	(0.42–1.32)
*Work status*									
Working	18.0			17.0			11.2		
Not working	20.6	1.14	(0.83–1.57)	17.6	1.02	(0.72–1.45)	13.9	1.44	(0.95–2.17)
*Self-rated health*									
Good	18.8			17.9			11.8		
Poor	14.0	0.84	(0.54–1.28)	9.8	0.66	(0.39–1.09)	9.1	0.96	(0.55–1.67)
*Psychological distress*									
No	21.2			21.0			14.6		
Yes	14.5	0.75	(0.59–0.94)	11.9	0.61	(0.48–0.79)	7.4	0.55	(0.39–0.77)

HL, health literacy; PR, prevalence ratio; 95% CI, 95% confidence interval.

Adjusted for all other variables in the table.

## Discussion

The present study examined the associations of combinations of general HL and COVID-19-related HL with COVID-19 protective behaviors and healthy lifestyle behaviors in Japan. High level in COVID-19-related HL only was associated with high adherence to COVID-19 protective behaviors, while high level in general HL only was associated with healthy and balanced diet, adequate sleep, and regular exercise.

High level in COVID-19-related HL only and high level in both HLs were associated with high adherence to COVID-19 protective behaviors. The situation during a newly emerging pandemic is typically characterized by urgency of actions, but is challenged by uncertain scientific knowledge and a high degree of complexity at all levels of action (31). Although the understanding of COVID-19 and its control are improving, there remains scientific uncertainty regarding many characteristics of the COVID-19 pandemic. Furthermore, COVID-19 is the first pandemic in history in which technology and social media are being used on a massive scale to keep people safe, informed, productive and connected (32). The technology we rely on to stay connected and informed is enabling and amplifying an infodemic—an overabundance of information—that continues to undermine the global response and jeopardizes measures to control the pandemic (32, 33). Therefore, it is a major challenge for individuals to integrate this sea of information into their personal behavioral actions (34). One review concluded that research should attempt to develop HL instruments that measure COVID-19-related HL (35). However, there are only a few COVID-19-related HL instruments (36, 37). One web-based study in Japan showing the association between high general HL measured by the Japanese version of the 47-item European health literacy survey questionnaire (HLS-EU-Q47) (38, 39) and COVID-19 protective behaviors stated that the assessment of HL may be inadequate to assess responses to pandemics caused by new viruses or infodemics because it may reflect the ability to cope with common diseases (16). One study found that domain-general and domain-specific abilities can influence the processing of information and subsequent adoption of behaviors distinctively (21). The present study showed that high level in COVID-19-related HL only, but not high level in general HL only, was associated with high adherence to COVID-19 protective behaviors. These findings indicate that general HL is insufficient for adherence to COVID-19 protective behaviors, whereas COVID-19-related HL is helpful in promoting adherence to COVID-19 protective behaviors even among individuals with low levels of general HL. Given this advantage of knowledge-based HL, it is speculated that optimal design of health messages is very important for supporting recipients’ comprehension of health information sufficiently to result in positive behavioral responses (40). Indeed, since the early phase of the COVID-19 pandemic, the Japanese government has used the following campaign slogan: “avoid the three Cs,” namely closed spaces with poor ventilation, crowded places with many people nearby, and close-contact settings such as close-range conversation. These clear messages regarding prevention of COVID-19 may partly explain the association between high level in COVID-19-related HL only and high adherence to COVID-19 protective behaviors.

High level in general HL only and high level in both HLs were associated with healthy lifestyle behaviors. Our finding is consistent with the finding of one study showing that high HL measured by the CCHL scale was associated with healthy lifestyles such as regular eating patterns and weekly exercise (25). It has been repeatedly reported that people with high HL are able to adequately understand and utilize health information in a way that protects and improves their health (41). High HL improves the health choices of individuals and their opportunities for certain health-related behaviors (42). The CCHL scale used in the present study explicitly attempts to assess critical HL in terms of information appraisal and ask respondents the extent to which they consider the reliability, validity, credibility and applicability of health information (25, 43). The present study also showed that high level in both HLs had the highest prevalences of high adherence to healthy lifestyle behaviors. Healthy lifestyle behaviors have been recommended since the start of the COVID-19 pandemic, because these behaviors affect the ability of the body to prevent, fight, and recover from infections and the stay-home request forces people to change their lifestyles (5). HL, as a social vaccine, acts as a means to understand and apply culturally appropriate and socially sensitive information about required protective behaviors to support the COVID-19 strategies of governments and health authorities (7). The combination of general HL and COVID-19-related HL can efficiently promote adherence to healthy lifestyle behaviors.

During the COVID-19 pandemic, concerns have repeatedly been raised about potential long-term damage to mental health (44). The present study showed associations between higher levels of HL and high adherence to COVID-19 protective behaviors as well as healthy lifestyle behaviors, even after adjustment for psychological distress. These findings indicate that the association between HL and adherence to protective behaviors cannot be explained by mental health alone. The present study further showed that psychological distress was associated with low adherence to healthy lifestyle behaviors, but not with adherence to COVID-19 protective behaviors. Different approaches are needed to promote adherence to COVID-19 protective behaviors and healthy lifestyle behaviors.

Participants with high level in both HLs were more likely to be men, be highly educated, and have higher income, and less likely to have psychological distress. Higher educational attainment is a strong precursor to higher general HL (45), which further leads to higher income status, especially in Japan, where education is a major factor affecting social stratification among men as bread winners, while women are less likely to undertake higher education (university bachelor’s degree or higher) (46). A higher level of general HL is reportedly associated with lower perceived risk of infection, higher compliance with appropriate protective behaviors, and lower risk of anxiety during the pandemic, even after adjusting for COVID19-specific knowledge (14).

The present findings have implications for promoting protective behaviors against COVID-19. Although vaccination delivery and roll-out have been in progress since 2021 in Japan, it is still possible to contract COVID-19 and spread it to others after being vaccinated. Therefore, people should continue to adhere to COVID-19 protective behaviors to keep themselves and others healthy. The observed associations of HL with COVID-19 protective behaviors and healthy lifestyle behaviors highlight the importance of HL for pandemic prevention and health maintenance. Because HL can be improved by education, it may be important to implement educational interventions during ordinary times, thus facilitating people to develop transferable skills in accessing, understanding, analyzing, and applying health information (47). However, it remains unclear how COVID-19-related HL can be improved. Therefore, governments and health authorities need to provide information on COVID-19 based on the level of COVID-19-related HL and also enhance general HL during ordinary times.

The present study has several limitations. First, the response rate was low. The participants of the J-SHINE survey in the first wave were comparable with the vital statistics of the target population in terms of age, sex, and educational attainment (22). However, the participants of the J-SHINE survey in the fourth wave were more likely to be older, be women, and highly educated than those who participated in the first wave but not in the fourth wave. Second, general HL was measured about 10 years before the COVID-19 pandemic. HL can change over time (48, 49), and one study showed that general HL declined significantly from immediately before the COVID-19 pandemic to 1 year later (47). However, the present study showed that high general HL was associated with healthy lifestyle behaviors, which is consistent with previous findings in Japan (25). Third, the reliability and validity of the questionnaire for COVID-19-related HL have not been verified. However, this questionnaire was adapted from validated questions (39). Fourth, the COVID-19 protective behaviors were self-reported, which is a source of uncertainty because participants may be influenced by social desirability bias.

In conclusion, the present study found that high level in COVID-19-related HL only was associated with high adherence to COVID-19 protective behaviors, while high level in general HL only was associated with healthy lifestyle behaviors. High level in both HLs showed the highest prevalence of high adherence to COVID-19 protective behaviors and healthy lifestyle behaviors. These findings indicate that consideration of both general HL and COVID-19-related HL could be useful for public health interventions to promote COVID-19 protective behaviors and maintain healthy lifestyle behaviors, with consequent preparation for possible future pandemics and public health crises.

## Data availability statement

The datasets presented in this article are not readily available because of ethical consideration under written consent with the survey respondents. Requests to access the datasets should be directed to the last author.

## Ethics statement

The Research Ethics Committee of The University of Tokyo, Graduate School of Medicine approved the survey procedure of the J-SHINE (Approval No. 3073). The J-SHINE Data Management Committee approved the secondary use of the data, with personally identifiable information deleted to ensure confidentiality. The studies were conducted in accordance with the local legislation and institutional requirements. The participants provided their written informed consent to participate in this study.

## Author contributions

KM conceived the study, performed the statistical analysis, and drafted the manuscript as the principal author. SK and HH provided advice regarding critically important intellectual content and helped to draft the manuscript. All authors contributed to the article and approved the submitted version.

## Funding

The J-SHINE survey was supported by a Grant-in-Aid for Scientific Research on Innovative Areas (grant number, 21119002) and a Grant-in-Aid for Scientific Research (A) (grant number, 18H04070) from the Ministry of Education, Culture, Sports, Science and Technology, Japan, and by a research grant from the Ministry of Health, Labour and Welfare, Japan (grant number, H27-Lifestyle-ippan-002). This work was supported by a Grant-in-Aid for Early-Career Scientists (grant number, 18K17397) and a Grant-in-Aid for Scientific Research (C) (grant number, 21K10490) from the Ministry of Education, Culture, Sports, Science and Technology, Japan. The funding sources played no role in study design, the collection, analysis and interpretation of data, the writing of the articles, or the decision to submit it for publication.

## Conflict of interest

KM is an employee of the Ministry of Education, Culture, Sports, Science and Technology, Japan.

The remaining authors declare that the research was conducted in the absence of any commercial or financial relationships that could be construed as a potential conflict of interest.

## Publisher’s note

All claims expressed in this article are solely those of the authors and do not necessarily represent those of their affiliated organizations, or those of the publisher, the editors and the reviewers. Any product that may be evaluated in this article, or claim that may be made by its manufacturer, is not guaranteed or endorsed by the publisher.
